# Reducing GEF-H1 Expression Inhibits Renal Cyst Formation, Inflammation, and Fibrosis via RhoA Signaling in Nephronophthisis

**DOI:** 10.3390/ijms24043504

**Published:** 2023-02-09

**Authors:** Qiulei Hu, Jiayong Lai, Huamu Chen, Yong Cai, Zhihui Yue, Hongrong Lin, Liangzhong Sun

**Affiliations:** 1 Department of Pediatrics, Nanfang Hospital, Southern Medical University, Guangzhou 510515, China; 2 Department of Pediatrics, The First Affiliated Hospital, Sun Yat-sen University, Guangzhou 510080, China

**Keywords:** nephronophthisis, GEF-H1, RhoA, cystogenesis, inflammation

## Abstract

Nephronophthisis (NPHP) is the most prevalent monogenic disease leading to end-stage renal failure in childhood. RhoA activation is involved in NPHP pathogenesis. This study explored the role of the RhoA activator guanine nucleotide exchange factor (GEF)-H1 in NPHP pathogenesis. We analyzed the expression and distribution of GEF-H1 in *NPHP1* knockout (*NPHP1*^KO^) mice using Western blotting and immunofluorescence, followed by GEF-H1 knockdown. Immunofluorescence and renal histology were used to examine the cysts, inflammation, and fibrosis. A RhoA GTPase activation assay and Western blotting were used to detect the expression of downstream GTP-RhoA and p-MLC2, respectively. In *NPHP1* knockdown (*NPHP1*^KD^) human kidney proximal tubular cells (HK2 cells), we detected the expressions of E-cadherin and α-smooth muscle actin (α-SMA). In vivo, increased expression and redistribution of GEF-H1, and higher levels of GTP-RhoA and p-MLC2 in renal tissue of *NPHP1*^KO^ mice were observed, together with renal cysts, fibrosis, and inflammation. These changes were alleviated by GEF-H1 knockdown. In vitro, the expression of GEF-H1 and activation of RhoA were also increased, with increased expression of α-SMA and decreased E-cadherin. GEF-H1 knockdown reversed these changes in *NPHP1*^KD^ HK2 cells. Thus, the GEF-H1/RhoA/MLC2 axis is activated in *NPHP1* defects and may play a pivotal role in NPHP pathogenesis.

## 1. Introduction

Nephronophthisis (NPHP) is an autosomal recessive cystic kidney disease that is the main genetic cause of end-stage renal disease in childhood [1,2,3,4]. Pathogenic genes encode proteins associated with the structure and function of the primary cilia. To date, over 25 pathogenic genes have been identified, the most prevalent of which is *NPHP1* [5,6,7,8,9]. Renal histopathological changes in NPHP are characterized by corticomedullary tubular dilation, cyst formation, diffuse interstitial fibrosis, and inflammation, as well as thickening and layering of the tubular basement membrane [10,11,12,13,14]. Importantly, the pathogenesis of NPHP is not yet fully understood [15]. RhoA activation and a perturbed cytoskeleton have been observed in a variety of ciliopathies, including NPHP, suggesting that RhoA may be implicated in the pathogenesis of NPHP [16,17]. However, the mechanism underlying RhoA activation in ciliopathies remains unclear.

RhoA belongs to the Rho GTPase family and functions as a binary switch regulated by guanine nucleotide exchange factors (GEFs) and GTPase-activating proteins (GAPs). GEFs activate RhoA by promoting the release of GDP and binding of GTP, whereas GAPs hydrolyze GTP to GDP and switch off RhoA to an inactive conformation [18]. RhoA activation initiates a large number of biological processes essential for cytoskeletal organization, cilium assembly, cell motility, proliferation, polarization, morphology, and contraction [19,20], as well as lumen formation, fibrosis, and inflammation [21,22,23].

Over 80 Rho GEFs have been identified. Human GEF-H1 and its murine homologue Lfc are members of the Dbl family, which has Dbl homology (DH) and pleckstrin homology (PH) domains [19,24]. GEF-H1 usually binds to microtubules and cell-cell junctions in quiescent cells, sequestering its interaction with RhoA. However, RhoA signaling is activated when microtubules are perturbed or GEF-H1 is phosphorylated [25,26,27,28,29,30]. The protein encoded by *NPHP1*, nephrocystin-1, which is located in the transition zone of the primary cilium and cell-cell junctions, is associated with microtubules and actin filament. Actin filament reorganization, delayed cell-cell junction formation, and disturbed cell polarity have been observed in mouse models and renal tubule cells with *NPHP1* defect [31,32,33].

RhoA activation mediated by GEF-H1 promotes nuclear translocation of the transcription factor zonula occludens 1 (ZO-1)-associated nucleic acid binding protein (ZONAB). GEF-H1 and ZONAB form a complex during this process, facilitating ZONAB’s nuclear translocation [34], which is involved in cell proliferation, differentiation, and lumen formation. In our previous study, we found that knockdown (KD) of *NPHP1* using small interfering RNA (siRNA) in MDCK cells induced nuclear translocation of ZONAB [35]. Therefore, we hypothesized that GEF-H1 may be involved in RhoA activation in diseases with *NPHP1* defects.

This study aimed to explore the role of the GEF-H1/RhoA pathway in NPHP pathogenesis. We found that the GEF-H1/RhoA pathway was activated in the kidney of *NPHP1* knockout (*NPHP1*^KO^) mice and *NPHP1* knockdown (*NPHP1*^KD^)HK2cells and that it was involved in cyst formation, fibrosis, and inflammation.

## 2. Results

### 2.1. The GEF-H1/RhoA Pathway Was Activated in the Renal Tissue of NPHP1^KO^ Mice and NPHP1^KD^ HK2 Cells

**Figure 1:**
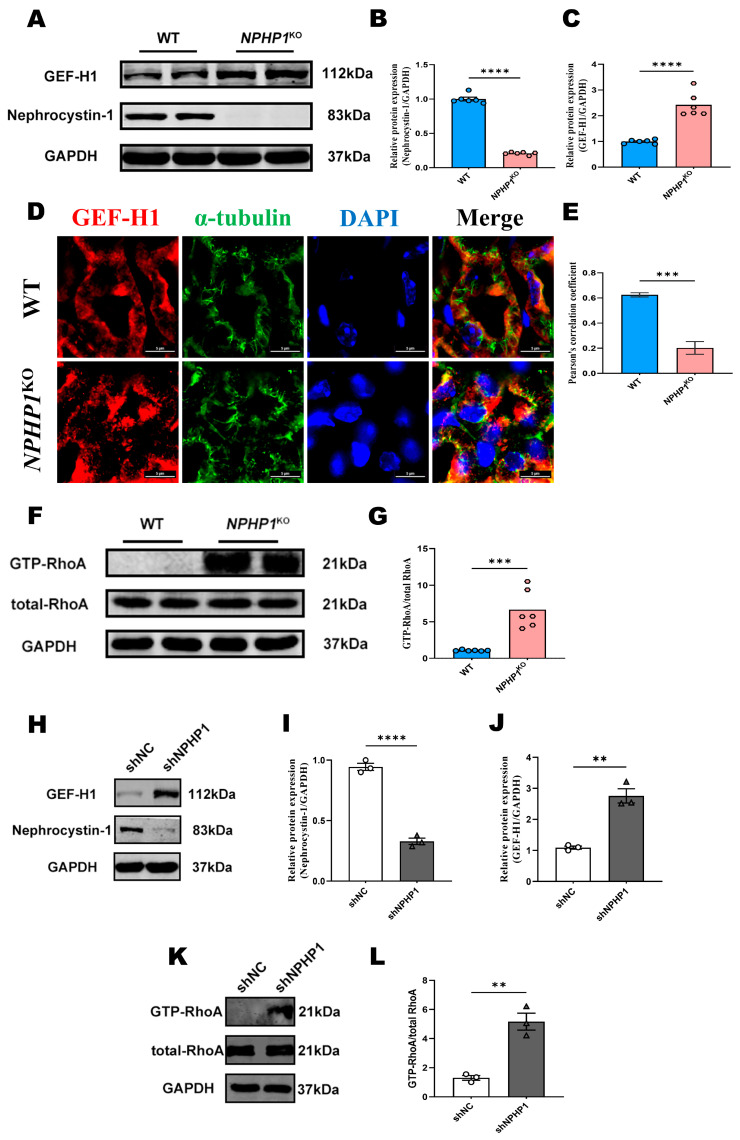
GEF-H1/RhoA pathway was activated in the renal tissue of *NPHP1*^KO^ mice and *NPHP1*^KD^ HK2 cells. (**A**–**C**): The expression of GEF-H1 was increased in the kidneys of *NPHP1*^KO^ mice compared with the wild-type (WT) mice by Western blot (**A**) and densitometry analysis (**B**,**C**), (*n* = 6 mice/group). Nephrocystin-1 was the protein encoded by *NPHP1* gene. (**D**): Intracellular spatial distribution of microtubules and GEF-H1 in WT and *NPHP1*^KO^ mice; In *NPHP1*^KO^ mice, GEF-H1 was more concentrated on the luminal side of the tubules (red), but the expression and intracellular distribution of microtubules remained unchanged (green) (Immunofluorescence, scale bar: 5 μm). (**E**): Pearson’s correlation coefficients showing the degree of colocalization of microtubules and GEF-H1 under different conditions (*n* = 6 mice/group). (**F**,**G**): GTP-RhoA were significantly upregulated in the kidney tissue of *NPHP1*^KO^ mice (**F**): Western blot, (**G**): Densitometry analysis, (*n* = 6 mice/group). (**H**–**J**): The expression of GEF-H1 was increased in *NPHP1*^KD^ HK2 cells (shNPHP1) compared with control cells (shNC) (**H**): Western blot, (**I**,**J**): Densitometry analysis, (*n* = 3). (**K**–**L**): GTP-RhoA was significantly upregulated in *NPHP1*^KD^ HK2 cells (shNPHP1) (**K**): Western blot, (**L**): Densitometry analysis, *n* = 3). Data represent the mean ± SEM. ** *p* < 0.01; *** *p* < 0.001; **** *p* < 0.0001; ns, no significance.

To investigate alterations in the GEF-H1/RhoA pathway in diseases caused by *NPHP1* gene defects, *NPHP1*^KO^ mice and *NPHP1*^KD^ HK2 cells were chosen for this study. Western blot analysis revealed that the expression of GEF-H1 increased in the kidneys of 24-week-old *NPHP1*^KO^ mice (Figure 1A–C). By immunofluorescence analysis, the intracellular distribution of GEF-H1 in tubular cells was altered, and GEF-H1 was more concentrated on the luminal side of the tubules in *NPHP1*^KO^ mice compared with the wild-type (WT) mice. The expression and intracellular distribution of microtubules remained unchanged, while Pearson’s correlation coefficients revealed that the degree of microtubule and GEF-H1 colocalization significantly decreased compared with WT mice (Figure 1D,E). Furthermore, downstream GTP-RhoA activity was elevated, indicating activation of the GEF-H1/RhoA pathway in *NPHP1*^KO^ mice (Figure 1F,G).

In vitro, *NPHP1*^KD^ HK2 cells were established following the successful transfection of lenti-shRNA. As shown by the Western blotting results, nephrocystin-1 (the protein encoded by the *NPHP1* gene) expression was significantly reduced (*p* < 0.0001), whereas the GEF-H1 levels were increased in *NPHP1*^KD^ HK2 cells (*p* < 0.01) (Figure 1H–J). Meanwhile, the expression of GTP-RhoA, the active form of RhoA, was increased (Figure 1K–L).

### 2.2. Knockdown of GEF-H1 Alleviated Renal Histological Injuries in NPHP1 ^KO^ Mice

**Figure 2:**
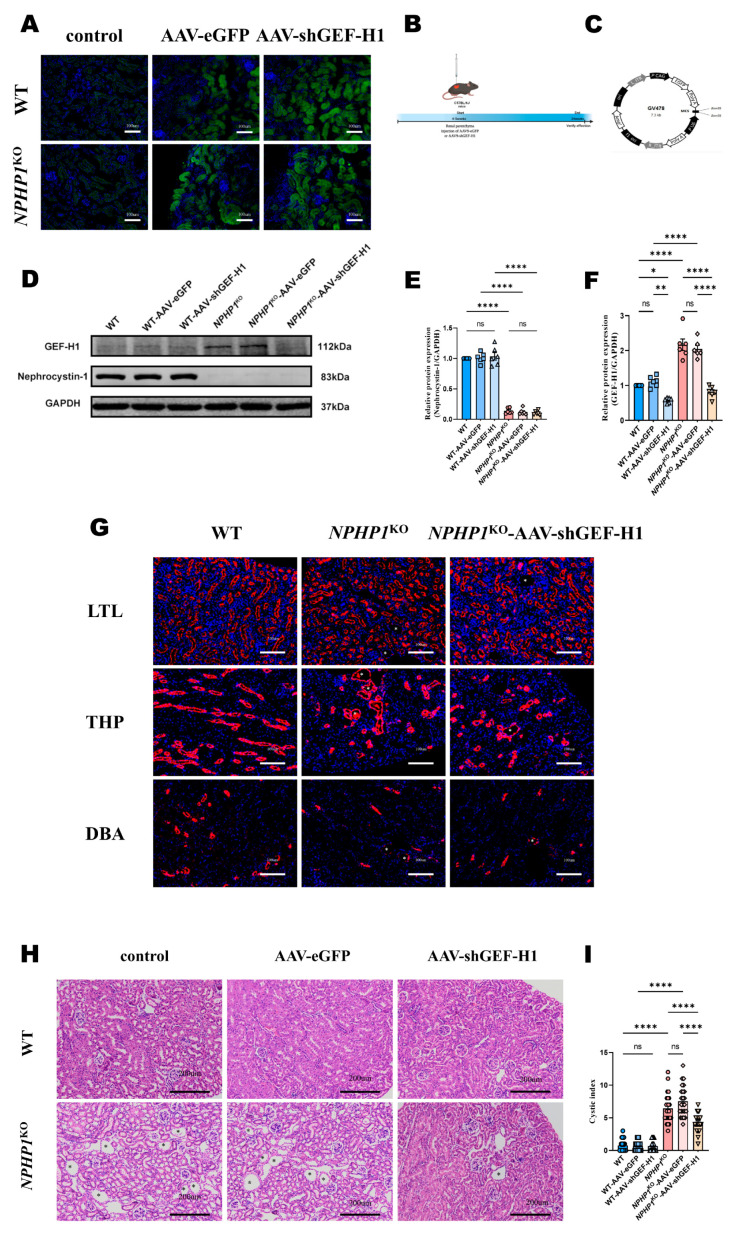
GEF-H1 knockdown reduced renal cyst formation in *NPHP1*^KO^ mice compared to WT mice. (**A**): Immunofluorescence analysis of adeno-associated virus (AAV) transfection efficiency. AAV-eGFP and AAV-shGEF-H1 transduced kidneys expressed eGFP (green). The sections were counterstained with DAPI (blue). (Light microscope, scale bar = 100 μm). (**B**): Time course of AAV9-mediated GEF-H1 knockdown. (**C**): Structural diagram of the AAV vector GV478. (**D**–**F**): The expression of GEF-H1 and nephrocystin-1 levels in mouse kidney tissue were evaluated by Western blot (**D**) and densitometry analysis. (**E**,**F**): Densitometry analysis; *n* = 6 mice/group). The increased GEF-H1 level of *NPHP1*^KO^ mice was then decreased after being transfected with AAV-shGEF-H1. (**G**): Staining of the kidney with lotus tetragonolobus lectin (LTL^+^ means proximal tubules; red), Tamm-Horsfall protein (THP+ means distal convoluted tubules; red), and Dolichus biflorus agglutinin (DBA+ means collecting ducts; red). Scale bar = 100 μm. (**H**): Mouse kidney histopathological changes (hematoxylin-eosin (HE) staining, scale bar = 200 μm). Asterisks indicate the cysts. Cyst formation and renal tubular dilatation in *NPHP1*^KO^ mice were alleviated after GEF-H1 knockdown. (**I**): Quantification of the cystic index in mouse kidney specimens (dots represent the number of cysts from 200× magnified images (4–5 images/mouse) (*n* = 6 mice/group). Data represent the mean ± SEM. * *p* < 0.05; ** *p* < 0.01; **** *p* < 0.0001; ns, no significance.

**Figure 3:**
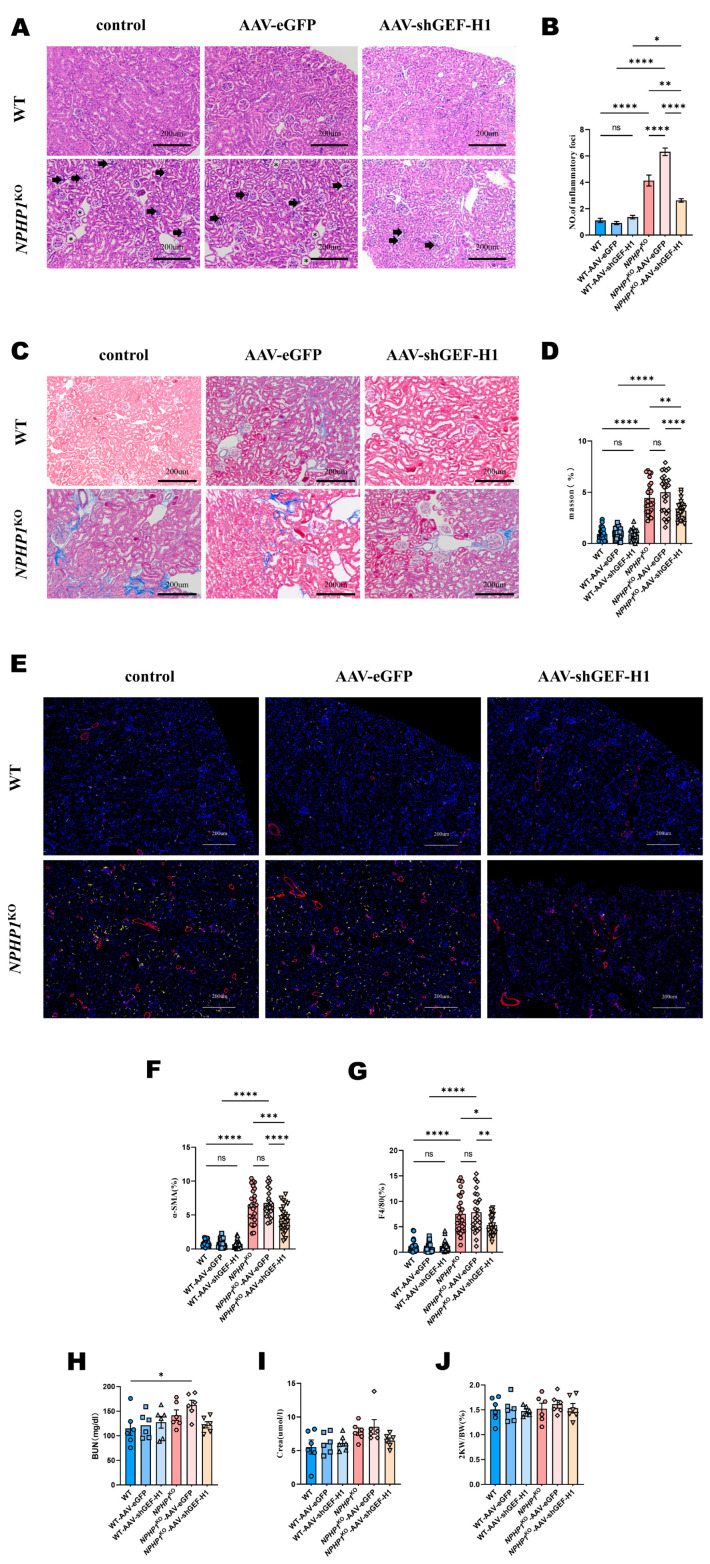
GEF-H1 knockdown alleviated renal interstitial inflammation and fibrosis in *NPHP1*^KO^ mice compared with WT mice. (**A**,**C**): Renal histology of mice from different groups. Asterisks indicate cysts, and black arrows indicate inflammatory cells. (**A**): HE staining, (**C**): Masson’s trichrome staining; scale bar = 200 μm). (**B**): Quantitative analysis of the number of inflammatory cells from a ×200 magnified image (8 images/mouse) (*n* = 6 mice/group). (**D**): Quantification of renal fibrosis by Masson staining of interstitial collagen. Each dot represents the percentage of staining of a ×200 magnified image (4–5 images/mouse) from *n* = 6 mice/group. Scale bar = 200 μm. (**E**): Immunostaining of the macrophage biomarker F4/80 (yellow) and myofibroblast biomarker α-smooth muscle actin (α-SMA, red). The increased F4/80 cell and α-SMA in the *NPHP1*^KO^ mice, which indicated renal interstitial inflammation and fibrosis, were then decreased after being transfected with AAV-shGEF-H1. Scale bar = 200 μm. (**F**,**G**): Quantification of F4/80 and α-SMA staining. Each dot represents the percentage of staining of a ×200 magnified image (4–5 images/mouse) from *n* = 6 mice /group. Scale bar = 200 μm. (**H)**: Blood urea nitrogen (BUN) level (*n* = 6 mice/group). (**I**): Creatinine (Crea) level (*n* = 6 mice/group). (**J**): Bilateral kidney weight (2KW) / body weight (BW) ratio (*n* = 6 mice/group). Data represent the mean ± SEM. * *p* < 0.05; ** *p* < 0.01; *** *p* < 0.001; **** *p* < 0.0001; ns, no significance.

To explore the effect of the GEF-H1/RhoA pathway on the pathogenesis of NPHP, adeno-associated virus-9 (AAV9) carrying shGEF-H1 or negative control vector (eGFP) was constructed and injected into the bilateral kidneys of mice at approximately 5 weeks, and the mice were sacrificed at 24 weeks (Figure 2B,C). Green eGFP was observed in the kidney tissues of mice transfected with AAV-shGEF-H1 and AAV-eGFP using immunofluorescence imaging (Figure 2A). Decreased GEF-H1 expression was detected in kidney specimens from the AAV9-shGEF-H1 transfected mice using Western blotting (Figure 2D–F). Cyst formation and renal tubular dilatation in the distal convoluted tubules (THP^+^) and collecting ducts (DBA^+^) were observed in *NPHP1* ^KO^ mice (Figure 2G). Renal cyst formation was alleviated (Figure 2G–I), and renal fibrosis and inflammation improved (Figure 3A,B) in GEF-H1 knockdown *NPHP1*^KO^ mice compared with control *NPHP1*^KO^ mice. The improvement in interstitial fibrosis and inflammation was further verified by comparing the amount of collagen deposition, fibroblasts, and macrophage infiltration in the kidney specimens of mice using Masson staining and immunostaining for α-smooth muscle actin and F4/80, respectively (Figure 3C–G). 

We observed a slight elevation in blood urea nitrogen and creatinine levels in *NPHP1*^KO^ mice compared with wild-type (WT) mice, which decreased with the AAV-shGEF-H1 intervention; however, these differences were not significant (Figure 3H–I). Differences in the bilateral kidney weight to body weight ratio (2KW/BW) of mice between the different groups were not significant (Figure 3J) (*p* < 0.05).

### 2.3. GEF-H1 Knockdown Alleviated Epithelial-Mesenchymal Transition (EMT) in NPHP1^KD^ HK2 Cells

**Figure 4:**
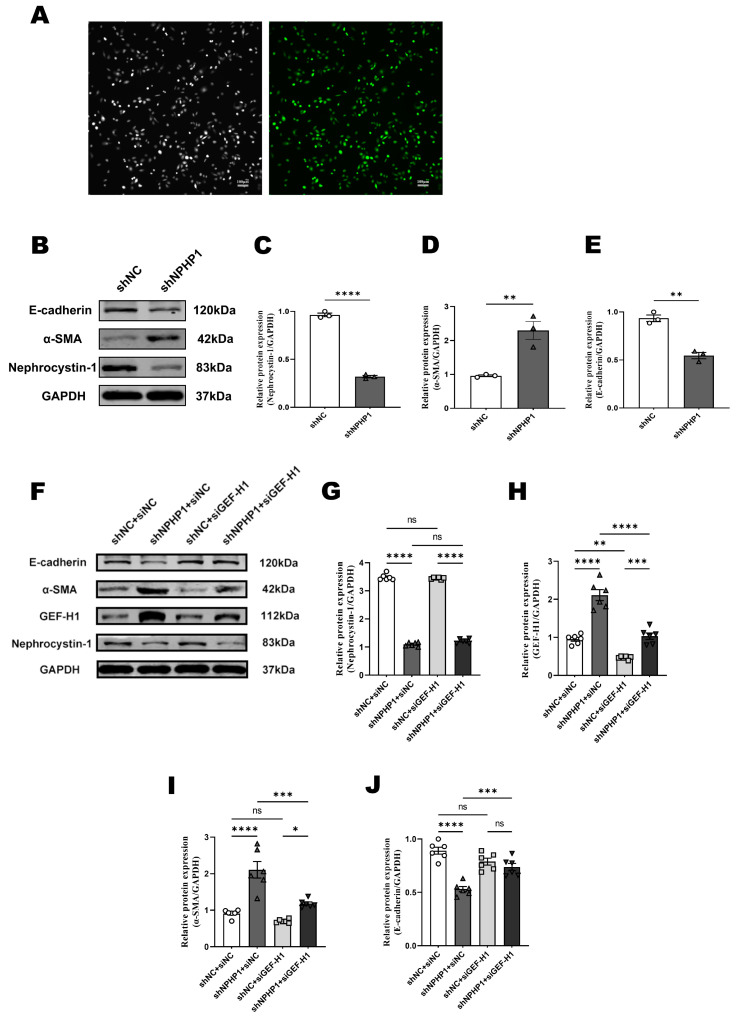
GEF-H1 knockdown alleviated epithelial-mesenchymal transition in *NPHP1*^KD^ HK2 cells. (**A**): eGFP was observed using fluorescence microscopy in HK2 cells transfected with lentivirus carrying shNPHP1. The left panels show the corresponding bright-field images of the cells. Scale bar = 100 μm. (**B**–**E**): Expression of nephrocystin-1, α-smooth muscle actin (α-SMA), and E-cadherin in HK2 cells (**B**): Western blot, (**C**–**E**): Densitometry analysis; *n* = 3 technical replicates). (**F**–**J**): Expression of GEF-H1, nephrocystin-1, α-SMA, and E-cadherin in HK2 cells (**F**): Western blot, (**G**–**J**): Densitometry analysis; *n* = 6 technical replicates. Data represent the mean ± SEM. * *p* < 0.05; ** *p* < 0.01; *** *p* < 0.001; **** *p* < 0.0001; ns, no significance.

We constructed the *NPHP1*^KD^ HK2 cell model by transfecting HK2 cells with lentivirus and visualizing eGFP under a fluorescence microscope (Figure 4A). Increased α-smooth muscle actin (α-SMA) expression and decreased E-cadherin expression in *NPHP1*^KD^ HK2 cells were detected using Western blotting compared with WT HK2 cells (Figure 4B–E), which indicated that *NPHP1*^KD^ HK2 cells were in the process of EMT.

To determine the effect of GEF-H1 knockdown on *NPHP1*^KD^ HK2 cells, we constructed a GEF-H1 knockdown siRNA (siGEF-H1) vector and a negative control vector (siNC). Compared with siNC-transfected cells, the expression of GEF-H1 protein was decreased in both *NPHP1*^KD^ and WT HK2 cells after siGEF-H1-transfected (*p* < 0.01). At 72 h after transfection with siGEF-H1, *NPHP1*^KD^ HK2 cells showed increased E-cadherin expression and decreased α-SMA expression (Figure 4F–J). These results indicated that GEF-H1 knockdown could reverse EMT in *NPHP1*^KD^ HK2 cells.

### 2.4. The GEF-H1/RhoA Pathway Was Associated with MLC2 Phosphorylation in NPHP1^KO^ Mice 

**Figure 5:**
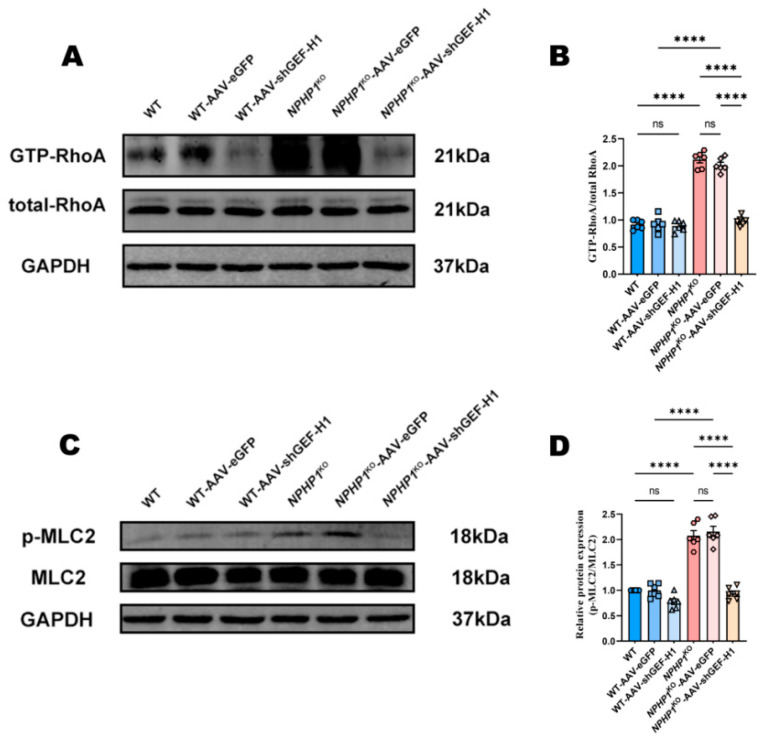
GEF-H1 knockdown blocked the activation of RhoA and the phosphorylation of MLC2 in *NPHP1*^KO^ mice. (**A**,**B**): The expression of RhoA activation in different mice groups. (**A**): Western blot, (**B**): Densitometry analysis; *n* = 6 mice/group). (**C**,**D**): The phosphorylation of MLC2(p-MCL2) in murine kidneys; p-MCL2 was upregulated in the kidneys of *NPHP1*^KO^ mice compared with that in WT mice. After GEF-H1 knockdown, this upregulation was decreased in *NPHP1* ^KO^ mice. (**C**): Western blot, (**D**): Densitometry analysis; *n* = 6 mice/group). Data represent the mean ± SEM. **** *p* < 0.0001; ns, no significance.

MCL2 is a downstream target of the GEF-H1/RhoA pathway and is a regulator of cell cytoskeletal reorganization, cell morphological changes, and inflammation [36]. To determine if MLC2 was activated in *NPHP1* defects, we measured the phosphorylation of MLC2 (p-MCL2) in the kidneys of *NPHP1*^KO^ mice. As expected, p-MCL2 was upregulated in the kidneys of *NPHP1* ^KO^ mice compared with WT mice. This upregulation was decreased after GEF-H1 knockdown in *NPHP1*^KO^ mice (Figure 5A–D). These trends are consistent with those observed for cysts, inflammation, and fibrosis. These results indicate that the GEF-H1/RhoA/MLC2 pathway may play an important role in the pathogenesis of NPHP.

## 3. Discussion

Our results show that the expression of GEF-H1 increased and that its intracellular distribution was altered in *NPHP1*^KO^ mice and *NPHP1*^KD^ HK2 cells. The expression and intracellular distribution of the microtubules remained unchanged. Pearson’s correlation coefficients revealed that the degree of microtubule and GEF-H1 colocalization significantly decreased. Downstream, RhoA was activated while the knockdown of GEF-H1 reversed these effects. These results indicated that not only did the expression of GEF-H1 increase but also that GEF-H1 detached from microtubules due to the defects of *NPHP1*. The GEF-H1/RhoA pathway was activated.

RhoA activation is tightly regulated by GEFs and GAPs. Aberrantly increased RhoA activity has been observed in many ciliopathies. However, the mechanisms underlying RhoA activation in ciliopathies remain largely unknown [37]. In autosomal dominant polycystic kidney disease (ADPKD), Streets et al. found that *PKD1* defects decreased the expression of centrosomal ARHGAP35, which is attributed to persistent RhoA activation and its downstream signals and associated cellular biological changes, such as actin cytoskeleton disorganization, ciliogenesis disturbance, and cell morphology changes [16].

*NPHP1* is a major pathogenic gene involved in NPHP. Its encoded protein, nephrocytin-1, normally resides in the transition zone of the cilium and cell-cell junctions. The mechanism by which *NPHP1* deficiency leads to GEF-H1 detachment from microtubules and is activated remains unclear and merits further study. GEF-H1 normally localizes to the microtubules through its N- and C-termini or through its PH domain in quiescent cells. GEF-H1 can also bind to microtubules by associating with other proteins, such as dynein motor light-chain Tctex-1 [29] and FAM123A, which interact with microtubule end-binding 1 and 3 proteins (EB1 and EB3) [38]. The binding of GEF-H1 to microtubules sequesters it and inhibits GEF activity on RhoA. GEF-H1 activation is usually achieved by microtubule depolymerization. However, depolymerization-independent activation of GEF-H1 has also been reported [38]. In this study, no apparent change in microtubules was observed, indicating that the activation of GEF-H1 in *NPHP1* defects is a depolymerization-independent mechanism.

The association between nephrocystin-1 and microtubules has been observed in previous studies. Otto et al. found that nephrocystin-1 interacts with β-tubulin both in vivo and in vitro. This interaction localizes at the C-terminal region between amino acid residues 237 and 670 of nephrocystin-1 [39]. Mollet et al. showed that nephrocystin-1 and nephrocystin-4 associate with α-tubulin in polarized and ciliated tubular MDCK cells [31]. These studies indicate that both α-tubulin and β-tubulin interact with nephrocystin-1. Defects in nephrocytin-1 may affect microtubules; however, more studies are needed to clarify this.

GEF-H1 can also bind to apical junctions (AJs) by integration with cingulin or paracingulin in polarized epithelial cells [31], which sequester it to interact with RhoA. Cell-cell junctions are also involved in the localization of nephrocystin-1. Therefore, nephrocystin-1 defects may also affect GEF-H1 in AJs. However, the association of nephrocytin-1 with GEF-H1 and its role in the pathogenesis of *NPHP1* defects deserves further study.

Our results revealed that the knockdown of GEF-H1 not only restored the activation of RhoA and phosphorylation of MCL2 but also reversed cyst formation, interstitial fibrosis, and inflammation in *NPHP1*^KO^ mice, and inhibited EMT changes in *NPHP1*^KD^ HK2 cells by downregulating the expression of α-SMA and upregulating the expression of E-cadherin. EMT may be involved in the pathogenesis of renal fibrosis in cilia-related cystic disease [40,41,42], as observed in kidneys from other NPHP patients and in mouse models [41]. Aberrant activation of RhoA, disorganized actin filaments, and disturbed ciliogenesis have long been observed in various ciliopathies [37]. Blocking of RhoA/ROCK signaling by the ROCK inhibitor Y-27632 restores cilia length and the disorganized actin filaments in *PKD1*^KD^ cells and reduces cyst formation in *PKD1*^KO^ mice [16]. Recently, Garcia H. et al. validated prostaglandin signaling as a target of *NPHP1*-defective NPHP. PGE1 treatment can decrease the upregulated RhoA activity and attenuate the renal manifestations in *NPHP1*^KO^ mice, including tubular dilatation, early upregulation of fibrosis markers and extracellular matrix components, and abnormal cilia [17].

Cyst formation, interstitial fibrosis, and inflammation are the characteristic pathological changes in NPHP. Cyst formation is a process influenced by many factors, such as cytoskeletal dynamics, intracellular and extracellular mechanical forces, and polarized membrane transport [43,44,45]. Crosstalk between different cytoskeletons is necessary to ensure a constant subcellular lumen diameter and to prevent cyst formation [45]. Cytoskeletal disturbances are common features of cyst formation. ROCK is a key actin-remodeling regulator responsible for the formation of stress fibers, and RhoA is an activator of ROCK [37]. Dysregulation of the RhoA/ROCK pathway leads to a highly disorganized cytoskeleton in cyst cells in ADPKD, as well as in other ciliopathies [16,37]. ROCK inhibitors have successfully improved cyst formation in ADPKD, suggesting that the cytoskeletal-associated RhoA/ROCK pathway may be a major factor in cyst initiation [16].

Fibrosis and inflammation are common pathophysiological processes that contribute to disease progression in chronic kidney diseases (CKDs). TGF-β plays a central role in renal fibrosis in most CKDs. In our previous study, we observed the activation of the TGF-β/Smad signaling pathway and its role in inducing EMT in *NPHP1*-defective MDCK cells [46]. Increased expression of TGF-β1 and EMT at the site of renal interstitial fibrosis were observed in *NPHP7*-mutant patients and mouse models [41]. However, GEF-H1 has been observed to be a downstream effector of TGF-β, and blockage of GEF-H1 can reverse TGF-β-induced cytoskeleton reorganization and EMT changes in vitro [47,48]. Inactivation of GEF-H1 in patients with mechanical trauma and uveitis can ameliorate disease progression by inhibiting fibrosis and inflammation [48]. 

In addition to actin remodeling, the GEF-H1/RhoA/MLC2 pathway is also implicated in mediating inflammation, intercellular permeability, and cell adhesion, which are associated with reperfusion injury, epithelial or endothelial barrier failure, and tumor metastasis [49,50,51].

In the present study, the knockdown of GEF-H1 abrogated RhoA and MLC2 activation in *NPHP1*^KO^ mice, with alleviated renal pathological changes, including cyst formation, interstitial fibrosis, and inflammation, as indicated by macrophage infiltration. In vitro, EMT changes in *NPHP1*^KD^ HK2 cells were reversed by GEF-H1 knockdown. These results indicate that the GEF-H1/RhoA/MLC2 axis plays a key role in the pathogenesis of *NPHP1*-defective NPHP. However, the detailed mechanism requires further investigation.

## 4. Materials and Methods

### 4.1. Mice and Antibodies 

*NPHP1*^KO^ *C57BL/6J* mice were generated by our group by deleting exons 2 to 20 of *NPHP1* using the CRISPR/Cas9 technique [52]. All animal experiments were performed according to the ARRIVE1 guidelines, and protocols were approved by the Institutional Animal Care and Use Committee of Nanfang Hospital, Southern Medical University. 

The following primary antibodies were used: anti-NPHP1 (Sigma Aldrich, Sab2104055, St. Louis, MO, USA), anti-GEF-H1 (CST, #4067, Danvers, MA, USA), anti-GAPDH (proteintech, 10494-1-AP, Rosemont, IL, USA), anti-RhoA (Proteintech, 10749-1-AP, Rosemont, IL, USA), anti-MLC2 (CST, #3672, Danvers, MA, USA), anti-pMLC2 (CST, #3671, Danvers, MA, USA), anti-F4/80 (Abcam, ab300421, Waltham, MA, USA), anti-α-SMA (CST, 19245S, Danvers, MA, USA), anti-E-cadherin (Santa Cruz, SC-1500, Dallas, TX, USA), and anti-α-tubulin (Immunoway, YM3035, Plano, TX, USA). Alexa-conjugated secondary antibodies (Invitrogen, Carlsbad, CA, USA) were also used.

### 4.2. AAV9 Injection in Situ

AAV9 carrying a recombinant GEF-H1-cDNA plasmid (AAV-shGEF-H1) or a control plasmid (AAV-eGFP) were constructed by Shanghai Genechem Company (Shanghai, China). The AAV9-U6-GEF-H1-CAG-EGFP vector was constructed to express shGEF-H1. AAV-shGEF-H1 was generated after cloning short-hairpin RNA (shRNA) fragments into the AAV vector GV478 (Figure 2C). The following primer sequence was used for expressing fragments of shGEF-H1: GCCCTCATTTGTCCTACATGT (Shanghai Genechem Co., Ltd., Shanghai, China). Five-week-old mice were immobilized with adhesive tape under intraperitoneal anesthesia with 1% sodium pentobarbital for surgery. We injected AAV-shGEF-H1 or AAV-eGFP from the back of mice into multiple kidney sites (6 × 10^10^ v.g/ kidney) using a microsyringe for experimental and control groups, respectively. We waited for virus diffusion for 15 s. The mice were randomly divided into six groups, including the WT group, WT-AAV-eGFP group, WT-AAV-shGEF-H1 group, *NPHP1*^KO^ group, *NPHP1*^KO^-AAV-eGFP group, and *NPHP1*^KO^-AAV-shGEF-H1 group. The AAV9-shGEF-H1 vector was validated to reduce GEF-H1 expression by Western blot (Figure 2D).

### 4.3. Urea and Creatinine Measurements 

Blood was collected from the retroorbital vein. The serum was then centrifuged and separated at room temperature. An automatic biochemical analyzer was used to detect urea and creatinine levels (Biobase BK280, Shandong, China). 

### 4.4. Cell Culture 

The HK2 cell line was purchased from the Public Medical Laboratory Center of Nanfang Hospital, Southern Medical University. The cells were routinely cultured and maintained at 37 °C in a humidified 5% CO2 incubator in DMEM/F12 (Gibco, Billings, MT, USA) containing 10% fetal bovine serum (Gibco, Billings, MT, USA) and 1% antibiotics (Gibco, Billings, MT, USA).

### 4.5. Lentiviral Transfection

For *NPHP1* knockdown, we used high-titer lentiviral shRNA vectors prepared by GenePharma Co. (Shanghai, China). Transfection was performed according to the protocol. Briefly, cells cultured in 24-well plates (1 × 10^4^ cells/well) were transduced with lentivirus for 24 h. Cells were then selected and expanded with puromycin (4 μg/mL) for 6 to 10 days after 48 h of transduction. The efficiency of infection was determined using Western blotting. We used a medium containing 2 μg/mL puromycin to establish stable knockdown (shNPHP1) or negative control (shNC) HK2 cell lines.

### 4.6. Transfection of siRNA 

HK2 cells were transfected with GEF-H1-specific siRNAs (GenePharma, Shanghai, China). The siRNA sequence used was si-GEF-H1:5′-CAGAUGUGUAAGACCUACUTT-3′. The negative control sequence (siNC) was 5′-UUCUCCGAACGUGUCACGUTT-3′. The siRNA-Mate reagent (GenePharma, Shanghai, China) was used to transfect cells according to the manufacturer’s instructions.

### 4.7. Protein Extraction and Western Blotting

Proteins were extracted from the tissue and cell samples and used for Western blot analysis. RIPA buffer (Beyotime, Shanghai, China) containing a protease inhibitor cocktail, and a phosphatase inhibitor cocktail was used to lyse the cells and tissues (CWBIO, Beijing, China). The amount and quality of proteins were determined using a bicinchoninic acid assay (BCA) (Beyotime, Shanghai, China) and a Synergy H1 Microplate Reader (BioTek, Shoreline, WA, USA). The diluted proteins were incubated at 100 °C for 5 min. Proteins were separated using 10% or 12% sodium dodecyl sulfate-polyacrylamide gel electrophoresis (SDS-PAGE) before being transferred to polyvinylidene difluoride membranes for Western blotting. The membranes were blocked with Tris-buffered saline supplemented with 0.1% (*v*/*v*) Tween 20 (TBST) and freshly-prepared 100 g/L non-fat milk for 2 h at 25 °C, followed by incubation at 4 °C overnight in the presence of various primary antibodies. The following day, the membranes were washed 3–5 times and incubated with fluorescein-conjugated secondary antibodies at room temperature for 1 h. An Odyssey scanner (Li-Cor Biosciences, Lincoln, NE, USA) was used to scan the immunoblots. Band intensities were quantified using the Image Studio software (Ver5.0, LI-COR, Lincoln, NE, USA), and GAPDH was used as the reference.

### 4.8. RhoA GTPase Activation Assay

GTP loading of RhoA was measured using a RhoA Activation Kit (NewEast Biosciences, Upper Merion Township, PA, USA) according to the manufacturer’s instructions. Briefly, a small fraction of the protein lysate was isolated and used to measure the total RhoA levels. The remaining protein lysate was incubated with glutathione S-transferase (GST) bound to Rhotekin RBD beads for 1.5 h. The beads were centrifuged, washed, and resuspended in a loading buffer. After boiling for 5 min, all samples were subjected to SDS-PAGE. Western blot analysis revealed the presence of RhoA.

### 4.9. Immunofluorescence 

Samples on glass slides were fixed for 20 min with ice-cold 4% paraformaldehyde, washed twice with phosphate-buffered saline (PBS), and permeabilized for 5 min with 0.1% Triton X-100 in PBS. Following a PBS wash, the cells were blocked with 10% goat serum for 1 h before incubation with primary antibodies overnight at 4 °C. Following 3 PBS washes, cells were incubated with secondary antibodies at room temperature for 1 h. Slides were mounted with an anti-fade reagent (Invitrogen SlowFade Gold) and photographed using a super-resolution microscope (N-SIM, Nikon, Tokyo, Japan) and an inverted microscope (OLYMPUS DP80, Tokyo, Japan).

### 4.10. Histopathological Analysis 

The kidney tissues were fixed in 4% paraformaldehyde, embedded in paraffin, and sectioned. Hematoxylin and eosin (HE), periodic acid-Schiff (PAS), and Masson’s trichrome staining were performed, and the results were observed under a light microscope. The ImageJ software(Ver1.8.0, NIH, Bethesd, Marylanda, USA) was used to analyze the HE and Masson trichrome-stained areas.

### 4.11. Statistical Analysis 

Experiments were independently repeated at least three times. Data were normally distributed, and a two-tailed *t*-test was used to compare the two groups. One-way analysis of variance (ANOVA) followed by the Tukey test was used for comparison among multiple groups. The data are presented as mean ± SEM. *p* < 0.05 was considered statistically significant. GraphPad Prism 9.3.0(Dotmatics, San Diego, CA, USA) was used for the statistical analysis.

## 5. Conclusions

Our results indicate that *NPHP1* defects induce GEF-H1 expression and alter its intracellular distribution. Altered GEF-H1 is a key regulator in the pathogenesis of *NPHP1*-defective NPHP, and GEF-H1-associated RhoA activation and MLC2 phosphorylation may play pivotal roles in the disease process. Our study provides novel insights into the pathogenesis of NPHP and a foundation for developing targeted treatments.

## Data Availability

All data generated and/or analyzed in this study are included in this published article.
